# Exploring the association between gestational diabetes exposure and mental and behavioural disorders in offspring: the Finnish gestational diabetes (FinnGeDi) register-based study

**DOI:** 10.1007/s00787-025-02800-y

**Published:** 2025-07-04

**Authors:** Jenni Kinnunen, Marja Vääräsmäki, Elina Keikkala, Sanna Mustaniemi, Eero Kajantie, Mika Gissler, Johan G. Eriksson, Risto Kaaja, Hannele Laivuori, Hilkka Nikkinen

**Affiliations:** 1 Research Unit of Clinical Medicine, Medical Research Center, University of Oulu, Oulu, 90029 Finland; 2 Wellbeing Services County of North Ostrobothnia, Department of Obstetrics and Gynaecology, Oulu University Hospital, Oulu, Finland; 3 Department of Public Health, Welfare Epidemiology and Monitoring Unit, Finnish Institute for Health and Welfare, Helsinki/Oulu, 00271/90101 Finland; 4 University of Helsinki and Helsinki University Hospital, Children’s Hospital, Helsinki, 00290 Finland; 5 Department of Clinical and Molecular Medicine, Norwegian University of Science and Technology, Trondheim, NO-7491 Norway; 6 Department of Data Analytics, Finnish Institute for Health and Welfare, Helsinki, 00271 Finland; 7 Research Centre for Child Psychiatry and Invest Research Flagship, University of Turku, Turku, 20014 Finland; 8 Academic Primary Health Care Centre, Region Stockholm, Stockholm, 11365 Sweden; 9 Department of Molecular Medicine and Surgery, Karolinska Institutet, Stockholm, 17176 Sweden; 10 Department of General Practice and Primary Health Care, Helsinki University Hospital, University of Helsinki, Helsinki, 00014 Finland; 11 Folkhälsan Research Center, Helsinki, 00250 Finland; 12 Department of Obstetrics and Gynecology, Human Potential Translational Research Programme, Yong Loo Lin School of Medicine, National University of Singapore, Singapore, 119228 Singapore; 13 Institute for Human Development and Potential (IHDP), Agency for Science, Technology and Research (A * STAR), Buona Vista, Singapore; 14 Institute of Clinical Medicine, Internal Medicine, Turku University Hospital, University of Turku, Turku, 20521 Finland; 15 Medical and Clinical Genetics, University of Helsinki and Helsinki University Hospital, Helsinki, Finland; 16 Institute for Molecular Medicine Finland, Helsinki Institute of Life Science, University of Helsinki, Helsinki, Finland; 17 Department of Obstetrics and Gynecology, Tampere University Hospital, The Wellbeing Services County of Pirkanmaa, Tampere, Finland; 18 Center for Child, Adolescent, and Maternal Health Research, Faculty of Medicine and Health Technology, Tampere University, Tampere, Finland

**Keywords:** Gestational diabetes, Offspring, Mental disorder, Behavioural disorder, Neurodevelopmental disorder

## Abstract

**Background:**

Gestational diabetes mellitus (GDM) is associated with an increased risk of attention deficit hyperactivity disorder (ADHD) and autism spectrum disorder (ASD) in offspring. Our aim was to investigate whether GDM exposure is linked to wider spectrum of mental and behavioural disorders in offspring during the first 10 years of life.

**Methods:**

This study included a population-based cohort of all women who delivered a singleton child in Finland in 2009, including 6,560 children exposed to maternal GDM and 51,770 control children. The main outcomes were the prevalence of mental and behavioural (including neurodevelopmental) disorders, and their subcategories, in study groups. Mother- and child-related covariates were adjusted for in the analyses.

**Results:**

Children exposed to GDM had a higher prevalence of mental and behavioural disorders (*n* = 1,010, 15.4%) compared with controls (*n* = 6,066, 11.7%; adjusted odds ratio (aOR): 1.18, 95% confidence interval [CI]: 1.09–1.28). In adjusted analyses, higher odds were observed only in boys (aOR: 1.25, 95% CI: 1.13–1.38). Specifically, GDM-exposed children had higher odds of behavioural disorders (aOR: 1.13, 95% CI: 1.02–1.25), developmental disorders (aOR: 1.14, 95% CI: 1.03–1.27) and behavioural disorders with physiological disturbances (aOR: 1.59, 95% CI: 1.16–2.18).

**Conclusions:**

Children exposed to maternal GDM have a higher prevalence of mental and behavioural disorders compared with non-exposed children. Notably, GDM exposure was shown to be an independent risk factor for these disorders in boys only.

## Background

Gestational diabetes mellitus (GDM) is a prevalent pregnancy-related disorder affecting 10–30% of pregnancies worldwide, and accounts for nearly 90% of diabetes cases in pregnancy [1–3]. It is defined as a glucose metabolism disorder detected for the first time during pregnancy, without meeting the criteria for type 1 or 2 diabetes [2, 3]. The prevalence of GDM has increased in recent decades due to increasing obesity and age among pregnant women, as well as more comprehensive GDM screening practices [4, 5].

Concurrently, mental and behavioural (including neurodevelopmental) disorders among children and adolescents have become more prevalent, affecting already 10–20% of children and adolescents worldwide [6–13]. Many of these disorders can become chronic and can cause long-term health burdens. Globally, they are among the major causes of morbidity, at substantial societal cost [11, 12, 14]. Additionally, many mental and behavioural disorders exhibit sex-specific prevalence and clinical manifestations [15, 16].

The association between maternal GDM and the risk of mental and behavioural disorders in offspring has been previously reported, as hyperglycaemia during pregnancy is known to impact fetal neurodevelopment [17–23]. Reactively elevated fetal insulin levels can modify fetal brain development, as insulin receptors are also present in the central nervous system [24, 25]. In addition, high glucose levels can mediate inflammation affecting neuronal integrity and disturb the neurodevelopment [25]. Furthermore, leptin, concentrations of which are more often elevated in GDM pregnancies, affects fetal hypothalamic development, which is involved in the regulation of behaviour and emotions, and the oxidative stress emphasised in these pregnancies alters fetal neurodevelopment [26–29]. Finally, epigenetic changes, modified by maternal metabolic environment, impact fetal neurodevelopment [30]. Meta-analyses by Zhao [31] and Wan [32] indicated that children exposed to GDM are at risk for attention deficit disorder (ADHD) and autism spectrum disorder (ASD), with Zhao [31] highlighting the risk for ADHD and Wan [32] focusing on ASD. However, important confounding factors, such as maternal body mass index (BMI) and socioeconomic status (SES), were not fully accounted for in previous studies [31, 32]. A subsequent meta-analysis by Rowland and Wilson (2021) confirmed the link between GDM exposure and increased ASD risk in offspring, but found no significant difference in ADHD risk, based on unadjusted analysis [33]. Overall, the scope of mental and behavioural disorders remains underexplored.

Another important yet underexplored issue is whether exposure to GDM has differential effects on the risk of mental and behavioural disorders according to offspring sex. Prior research has indicated that the intrauterine environment influences boys and girls differently, although the biological mechanism underlying this difference is not fully understood [29, 34–36].

Our aim was to investigate the association between maternal GDM and the risk of mental and behavioural disorders in offspring at 10 years of age in a Finnish register- and population-based cohort, and to examine potential sex-differences in the effect of GDM exposure.

## Materials and methods

**Fig. 1:**
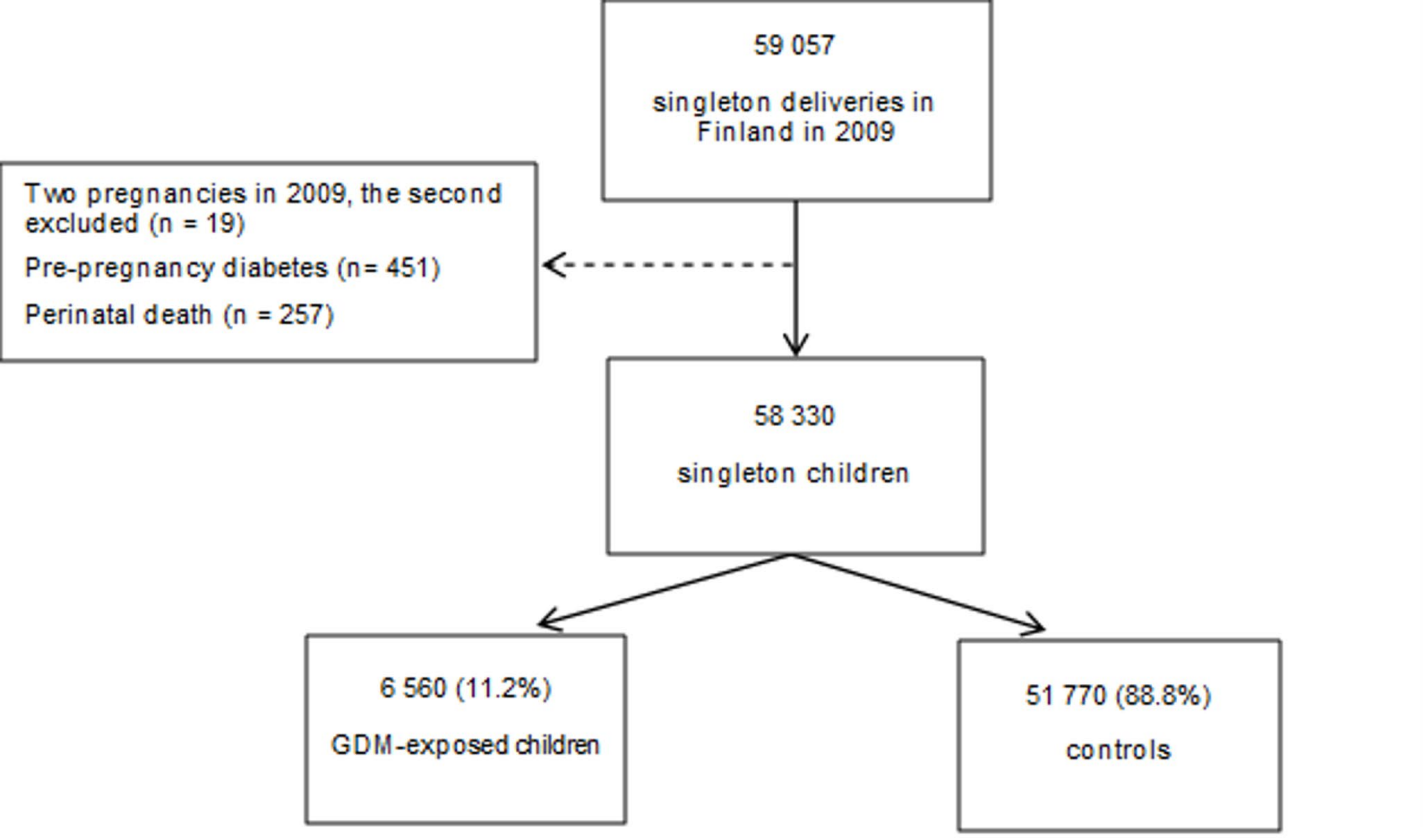
Flowchart of the study population. Perinatal death = intrauterine death ≥ 22 weeks of gestation or death in the first week of life

This study stemmed from the register-based arm of the Finnish Gestational Diabetes (FinnGeDi) study, which was established in 2009 after a new national comprehensive GDM screening took effect in 2008. The study protocol has been described previously in detail [37]. The study cohort was identified from the Finnish Medical Birth Registry (MBR) and included all singleton pregnancies in Finland in 2009 (*n* = 59,057). For women who had two deliveries in 2009, the child born from the second pregnancy was excluded (*n* = 19). Additionally, children of mothers with pre-existing diabetes (O24.0, O24.1, E10, E11) (*n* = 451) were excluded, as were perinatal deaths (*n* = 257). Mother–child data pairs were linked using personal identification numbers, which were pseudonymised by an individual not involved in the study. Ultimately, a total of 58,330 children were included in the study: 6,560 children who were exposed to GDM, and 51,770 children who served as controls. Flowchart shown in Fig. 1.

The data for this study were obtained from the MBR and the Finnish Care Register for Health Care (FCR), both maintained by the Finnish Institute for Health and Welfare (THL). The MBR includes all live births and stillbirths in Finland with at least 22 weeks of gestation or with a birth weight of ≥ 500 g, along with information related to pregnancy, delivery and the perinatal period. The FCR contains all inpatient hospital stays since 1969 and outpatient hospital treatments by physicians in specialised care since 1998, with the diagnoses coded according to the International Statistical Classification of Diseases and Related Health Problems 10th revision (ICD-10) since 1996, according to the 9th revision (ICD-9) in 1987–1995, and according to the 8th revision (ICD-8) in 1969–1986. The FCR is well validated for psychiatric diagnoses, and the MBR is both comprehensive and well validated [38, 39].

The FinnGeDi study protocol was approved by the Regional Ethics Committee in Northern Ostrobothnia Hospital District and by the THL. Permission for access to the registry data for the study was provided by the registry administrator of the THL. As the study was register-based, the study population was not contacted, as no permission was required from the population for their data.

### Screening and definition of GDM

Comprehensive screening of GDM in Finland was performed in 2009 following the new Finnish Current Care Guidelines. The guidelines recommend an oral glucose tolerance test (OGTT) for all pregnant women at 24–28 weeks of gestation, except for normal-weight (BMI 18.5–25.0 kg/m^2^) nulliparous women under 25 years of age and normal-weight multiparous women under 40 years of age with no prior GDM or macrosomic newborns. High-risk mothers (a history of GDM, a previous macrosomic child [birth weight ≥ 4,500 g], obesity [BMI ≥ 35.0 kg/m^2^], glucosuria, a family history of type 2 diabetes, use of systemic corticosteroid medication or polycystic ovary syndrome) were recommended to undergo OGTT at 12–16 weeks and, if the results were normal, to undergo OGTT again at 24–28 weeks. OGTT was also recommended when GDM was clinically suspected at any gestational week. OGTT is performed after an overnight fast, using a 75 g glucose load, with cut-off values of venous plasma glucose ≥ 5.3 mmol/l fasting, ≥ 10.0 mmol/l at one hour, and ≥ 8.6 mmol/l at two hours. A GDM diagnosis is based on one or more abnormal OGTT values. Following diagnosis, blood glucose levels are self-monitored, with target values of < 5.5 mmol/l fasting and < 7.8 mmol/l one hour postprandial. Women diagnosed with GDM receive dietary and lifestyle counselling, and pharmacological treatment, most often insulin, is initiated if target values are repeatedly exceeded [40]. 

In this study, a GDM diagnosis was established if the MBR included information on at least one of the following: abnormal OGTT result (yes/no), initiation of insulin treatment during pregnancy (yes/no) or a diagnosis of GDM (ICD-10: O24.4 or O24.9). The MBR has been validated to detect GDM with an accuracy of 94.2% [37]. Background data for the mothers and children were obtained from the MBR.

### Definition of mental and behavioural disorders in children

Diagnoses of mental and behavioural disorders in children were recorded in the FCR from birth up to 10 years of age. These disorders were analysed collectively (any mental, behavioural or neurodevelopmental disorder, ICD-10: F00–F99) and categorised into subcategories according to the ICD-10 classification presented in Table 1. No subcategories were excluded from the overall analysis of these disorders. However, subcategories that are rare or typically do not manifest before the age of 10 were not analysed separately: F00–F09 (organic, including symptomatic mental disorders, e.g. dementia), F10–F19 (mental and behavioural disorders due to psychoactive substance use, e.g. alcohol use), F20–F29 (schizophrenia), F30–F39 (mood disorders) and F60–F69 (disorders of adult personality and behaviour).

**Table 1:** Definitions of the subcategories of mental and behavioural disorders (ICD-10 codes: F00-F99) in children as examined in the study

ICD-10 Code	Abbreviation	Disorder
F40–F48	Anxiety disorders	Anxiety, stress-related, somatoform and other nonpsychotic disorders
F50–F59	Behavioural disorders with physiological disturbances	Behavioural syndromes associated with physiological disturbances
F70–F79	Intellectual disorders	Intellectual disabilities
F80–F89	Developmental disorders	Pervasive and specific developmental disorders *incl. ASD F84*
F90–F98	Behavioural disorders	Behavioural and emotional disorders *incl. ADHD and conduct disorders F90–F92*

ICD-10 codes = International statistical classification of diseases and related health problems 10th revision codes, ADHD = attention deficit and hyperactivity disorder, ASD = autism spectrum disorder

### Other definitions

Gestational age at delivery and birth weight were combined into a variable of perinatal adverse outcome (no/yes), which included preterm birth (< 37 + 0 weeks of gestation) or small for gestational age (SGA), defined as a birth weight or length of more than two standard deviations (SDs) below the sex and gestational age-specific mean. Large for gestational age (LGA) was defined as a birth weight or length of more than two SDs above the sex and gestational age-specific mean [41]. Maternal hypertensive disorders were identified using ICD-10-codes I10, O10, O13, O14 and O15. Maternal mental and behavioural disorders were retrieved from the MBR and the FCR using ICD-10 codes F00–F99, as well as ICD-9 and ICD-8 codes (280–319) from 1987 to 2019.

### Statistics

Statistical analyses were performed using IBM SPSS Statistics 29.0 (IBM SPSS Statistics for Windows, Version 29.0, Armonk, NY: IBM Corp.). Differences in pregnancy and child characteristics between groups were analysed using a Student’s *t*-test for continuous variables and an *χ*^2^-test for categorical variables to assess frequencies and evaluate differences within the population. Logistic regression analysis was conducted to compare the prevalence of mental and behavioural disorders in children between groups. Univariate analysis was used to identify potential confounders for significant variables for the adjustment model. Categorical variables were dummy coded for the analysis. We also assessed multicollinearity among the selected independent variables, with variance inflation factors ranging from 1.0 to 2.0. Interaction terms between GDM and offspring sex were created to investigate potential differential effects of GDM exposure on boys and girls.

## Results

Women with GDM were older and more likely to be obese and multiparous than those without GDM. They had a lower SES, smoked more often and had higher rates of hypertensive and mental health disorders. Children exposed to GDM were more likely to be born preterm and/or via caesarean section, and they were more often LGA and less likely to be SGA than were unexposed children. Demographic characteristics of the children and mothers are presented in Table 2.

**Table 2:** Demographic characteristics of the mothers and children within the groups

	GDM-pregnancies (*n* = 6 560)	Controls (*n* = 51 770)				
	*n* (%) / Mean (SD)	*n* (%) / Mean (SD)				*p*-value
Maternal age at delivery in years	31.1 (5.6)	29.3 (5.3)				< 0.001
< 20	107 (1.6%)	1 277 (2.5%)				
20–24	699 (10.7%)	8 511 (16.4%)				
25–29	1 791 (27.3%)	16 862 (32.6%)				
30–34	2 114 (32.2%)	16 671 (32.2%)				
35–39	1 334 (20.3%)	6 856 (13.2%)				
40-44	491 (7.5%)	1541 (3.0%)				
≥ 45	24 (0.4%)	52 (0.1%)				
Maternal pre-pregnancy BMI kg/m^2^	28.4 (±6.0)	23.7 (±4.3)				< 0.001
< 18.5	83 (1.3%)	2 009 (4.0%)				
18.5–24.9	1 994 (30.4%)	33 760 (67.3%)				
25–29.9	2 077 (31.7%)	10 082 (20.1%)				
30–34.9	1 381 (21.1%)	3 098 (6.2%)				
≥ 35	881 (13.4%)	1 206 (2.4%)				
Missing	144 (2.2%)	1 615 (3.1%)				
Parity						
1	2 386 (36.3%)	22 269 (43.0%)				< 0.001
2–3	3 307 (50.4%)	24 709 (47.7%)				< 0.001
≥ 4	867 (13.2%)	4 792 (9.3%)				< 0.001
Socioeconomic status						< 0.001
Higher official	1 016 (15.5%)	9 082 (17.5%)				
Lower official	2 270 (34.6%)	16 423 (31.7%)				
Manual worker	997 (15.2%)	6 406 (12.4%)				
Other	992 (15.1%)	8 590 (16.6%)				
Missing	1 285 (19.6%)	11 269 (21.8%)				
Smoking						< 0.001
No	5 249 (80.0%)	42 673 (82.4%)				
Cessation during first trimester	427 (6.5%)	2 542 (4.9%)				
Continued throughout pregnancy	746 (11.4%)	5 223 (10.1%)				
Missing	138 (2.1%)	1 332 (2.6%)				
Maternal mental disorder						
Before delivery	834 (12.7%)	5 823 (11.2%)				< 0.001
Diagnosed after delivery	642 (9.8%)	4436 (8.6%)				< 0.001
No	5 089 (77.5%)	41 599 (80.2%)				< 0.001
Maternal hypertensive disorder						< 0.001
Yes	673 (10.3%)	2 658 (5.1%)				
No	5 887 (89.7%)	49 112 (94.9%)				
Mode of delivery						< 0.001
Vaginal	5 151 (78.5%)	44 381 (85.7%)				
Caesarean	1 409 (21.5%)	7 386 (14.3%)				
Missing	0 (0%)	3 (0%)				
Perinatal outcomes						
Gestational age at delivery (weeks)	39.6 (1.6)	39.9 (1.6)				0.002
Preterm birth < 37 weeks	324 (4.9%)	1 990 (3.8%)				< 0.001
Term birth 37+0–41+6 weeks + days	5 970 (91.1%)	46 832 (90.6%)				0.156
Post-term birth ≥42 weeks	260 (4.0%)	2 875 (5.6%)				< 0.001
Missing	6 (0.1%)	73 (0.1%)				0.304
Birth weight (g)	3 607 (538.1)	3 509 (521.2)				< 0.001
SGA	143 (2.2%)	1 617 (3.1%)				< 0.001
LGA	294 (4.5%)	873 (1.7%)				< 0.001
Perinatal adverse outcome						0.615
Yes	439 (6.7%)	3 380 (6.5%)				
No	6 121 (93.3%)	48 390 (93.5%)				
Offspring’s sex (f/m)	3 202/3358	25 363/26 407				0.783

Socioeconomic status based on maternal occupation, other including self-employed individuals, stay-at-home mothers, students and pensioners; Perinatal adverse outcome = preterm birth < 37 weeks or SGA = small for gestational age ≤ two standard deviations below the sex and gestational age-specific mean, LGA = large for gestational age ≥ two standard deviations above the sex and gestational age-specific mean. *P*-values are based on student’s t-test in case of linear variables and on χ^2^-test in case of categorical variables

### Association between GDM and mental and behavioural disorders in children

Mental or behavioural disorders were diagnosed in 12.1% (*n* = 7,076) of the children. Among GDM-exposed children, the rate was 15.4% *(n* = 1,010), compared to 11.7% (*n* = 6,066) in the control group (odds ratio [OR]: 1.37, 95% confidence interval [CI]: 1.28–1.47, *p* < 0.001). This association remained significant after adjusting for confounding factors (adjusted odds ratio [aOR]: 1.18, 95% CI: 1.09–1.28, *p* < 0.001). We also analysed insulin-treated (*n* = 965, 14.7%) and diet-treated (*n* = 5,595, 85.3%) GDM separately. Both groups had higher odds of the examined disorders compared with unexposed children (insulin-treated aOR: 1.34, 95% CI: 1.12–1.60, *p* = 0.001; diet-treated aOR: 1.18, 95% CI: 1.09–1.29, *p* < 0.001).

### Sex differences in mental and behavioural disorders in children exposed to GDM

**Fig. 2:**
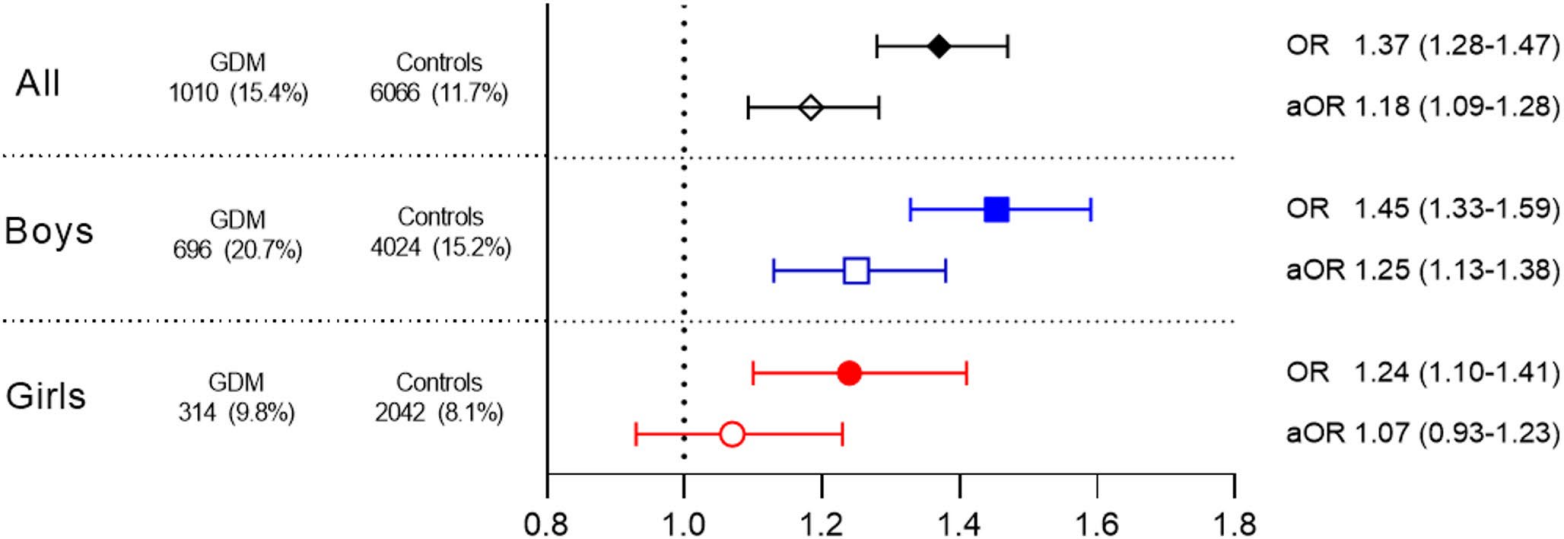
Odds ratios (ORs) and adjusted odds ratios (aORs) for any mental or behavioural disorder (F00–F99) in GDM-exposed boys and girls at 10 years of age. GDM = gestational diabetes

Mental and behavioural disorders were more common in boys (16.0%) than girls (8.2%). The interaction term between GDM exposure and sex concerning all these disorders was significant (*p* = 0.044). Among boys, the prevalence of disorders was higher in the GDM-exposed group (20.7%) than in the control group (15.2%, OR: 1.45, 95% CI: 1.33–1.59, *p* < 0.001), and this difference persisted after adjustments (aOR: 1.25, 95% CI: 1.13–1.38, *p* < 0.001). Among girls, 9.8% of the GDM-exposed and 8.1% of the controls had a diagnosis of mental or behavioural disorder (OR: 1.24, 95% CI: 1.10–1.41, *p* < 0.001). In girls the difference between groups was statistically insignificant in adjusted analysis (aOR: 1.07, 95% CI: 0.93–1.23, *p* = 0.348; Fig. 2).

### Differences in the subcategories of mental and behavioural disorders between the groups

Behavioural and developmental disorders were the most prevalent subcategories, with prevalence rates of 7.1% (*n* = 4,118) and 6.5% (*n* = 3,820). Behavioural disorders were diagnosed in 8.5% of the GDM-exposed children and 6.9% of the controls (OR: 1.41, 95% CI: 1.28–1.54, *p* < 0.001), while developmental disorders were diagnosed in 8.6% of the GDM-exposed children and 6.3% of the controls (OR: 1.26, 95% CI: 1.14–1.38, *p* < 0.001). The differences remained significant after adjustments (aOR: 1.13, 95% CI: 1.02–1.25, *p =* 0.022 and aOR: 1.14, 95% CI: 1.03–1.27, *p =* 0.011, respectively). We also analysed ADHD and conduct disorders (ICD-10: F90–F92), as well as ASD (ICD-10: F84), separately. The prevalence of ADHD and conduct disorders was 4.7% (*n* = 310) in GDM-exposed children and 3.7% (*n* = 1,950) in the controls (OR: 1.27, 95% CI: 1.12–1.43, *p* < 0.001), whereas the prevalence of ASD was 1.4% (*n* = 94) in GDM-exposed children and 1.0% (*n* = 507) in the controls (OR: 1.47, 95% CI: 1.18–1.84, *p* < 0.001). However, the differences in these specific disorders between the groups were attenuated after adjustments (ADHD and conduct disorders: aOR: 1.04, 95% CI: 0.91–1.19, *p* = 0.555; ASD: aOR: 1.19, 95% CI: 0.93–1.51, *p* = 0.160).

**Fig. 3:**
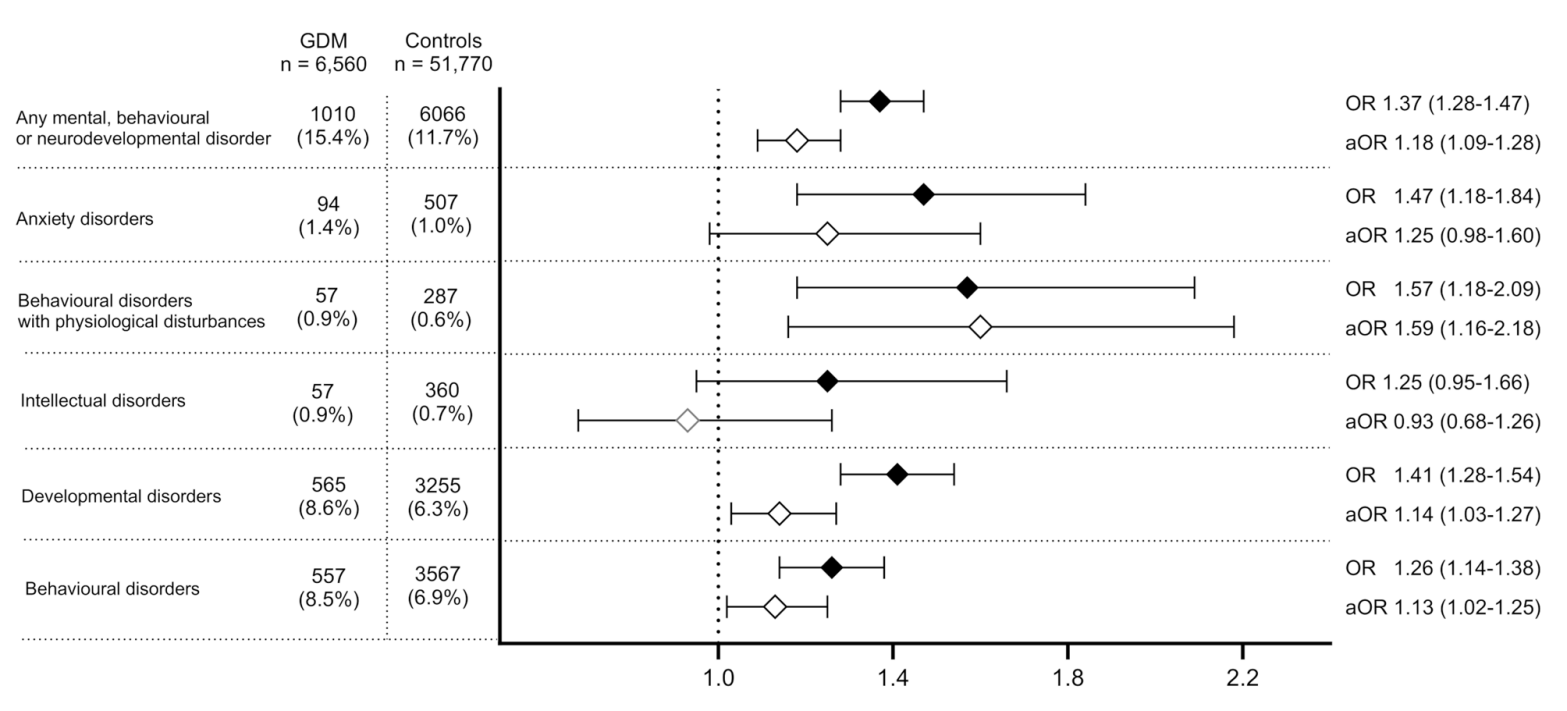
Mental and behavioural disorders and their subcategories in GDM-exposed children compared with controls up to the age of 10 years. GDM = gestational diabetes mellitus, OR = odds ratio, aOR = adjusted odds ratio, and p = *p*-value. Adjusted for maternal age, pre-pregnancy body mass index (BMI), parity, smoking, mother’s mental health disorder, mother’s hypertensive disorder and socioeconomic status (SES), mode of delivery, any perinatal adverse outcome, and offspring sex. Any mental, behavioural or neurodevelopmental disorder, ICD-10 F00–F99; anxiety disorders, ICD-10 F40–F48; behavioural disorders with physiological disturbances, ICD-10 F50–F59; intellectual disorders, ICD-10 F70–F79; developmental disorders, ICD-10 F80–F89; behavioural disorders, ICD-10 F90–F98

Anxiety disorders were diagnosed in 1.0% (*n* = 601) of the children: 1.4% (*n* = 94) in GDM-exposed children, and 1.0% (*n* = 507) in the controls (OR: 1.47, 95% CI: 1.18–1.84, *p* < 0.001). However, adjustment for confounding factors attenuated this association (aOR: 1.25, 95% CI: 0.98–1.60, *p =* 0.073). Behavioural disorders with physiological disturbances were diagnosed in 0.6% (*n* = 344) of the children, with 0.9% (*n* = 57) in GDM-exposed children and 0.6% (*n* = 287) in the controls (OR: 1.57, 95% CI: 1.18–2.09, *p =* 0.002). Concerning these disorders, GDM was revealed to be an independent risk factor (aOR: 1.59, 95% CI: 1.16–2.18, *p* = 0.004). The prevalence of intellectual disorders was 0.9% (*n* = 57) in GDM-exposed children and 0.7% (*n* = 360) in the controls, with no statistical difference between the groups (OR: 1.25, 95% CI: 0.95–1.66, *p* = 0.117). Results are shown in Fig. 3. Interaction terms between GDM exposure and sex in the subcategories of the disorders were non-significant (*p* > 0.05), and subcategory analyses were not stratified by sex.

## Discussion

In our population-based study, offspring exposed to maternal GDM were more likely to be diagnosed with mental and behavioural disorders at 10 years of age compared to unexposed children. This difference was observed after multiple adjustments. When stratified by sex, GDM was an independent risk factor for these disorders, but only in boys. Higher rates of behavioural disorders, developmental disorders and behavioural disorders with physiological disturbances were observed in GDM-exposed children compared to controls, despite the multiple confounders considered. However, when analysing specific disorders, such as ADHD and conduct disorders (F90–F92), and ASD (F84) separately, they were more prevalent in GDM-exposed children, but there was no difference between the groups after the confounders were considered.

Our study comprehensively examined mental and behavioural disorders. Although the results of previous research are in line with our findings, there are currently no fully comparable studies in this area. For instance, in a Finnish study focusing on the effect of both pre-existing and gestational diabetes, maternal pre-existing diabetes exposure was a stronger risk factor for these disorders than GDM. Additionally, higher maternal BMI was more strongly associated with the risk of these disorders in offspring than GDM, with hazard ratios ranging from 1.12 to 1.66 depending on the mother’s weight status [42]. Although maternal pre-existing diabetes has been shown to have a more pronounced impact on child neurodevelopment than GDM, it is crucial to recognise that maternal hyperglycaemia during pregnancy is most often due to GDM [2]. A salient feature of the intrauterine environment in pre-existing diabetes and GDM is the onset of hyperglycaemia and fetal hyperinsulinemia, which, in GDM, typically does not occur until the second trimester [43]. A possible mechanism by which impaired glucose metabolism may lead to neurodevelopmental problems in offspring is the presence of insulin hormone receptors in the central nervous system [26]. Furthermore, elevated leptin concentrations in GDM pregnancies influence fetal hypothalamic development, which regulates behaviour and emotions [44–46]. Moreover, oxidative stress, chronic inflammation and elevated fatty acid levels, which are prominent in GDM pregnancies, may induce epigenetic changes and have long-term effects on fetal neurodevelopment [29, 30, 47–51].

The overall prevalence of the mental and behavioural disorders examined in our study was higher in boys, a finding which is consistent with previous literature [9, 52]. The interaction term indicated that the risk of these disorders in GDM-exposed children differed by sex, and, in the adjusted analysis, the risk was observed only in boys. Previous research has identified mechanisms that may underlie the sex-specific effects of GDM exposure. Insulin regulates placental function, and impairment in the signalling pathway in the placenta has been shown to particularly affect the development of male offspring in ways that are common to neurodevelopmental disorders [34, 36]. Although the difference between sexes might be at least partly due to differential effects of GDM, it also likely to be related to the fact that boys are more often diagnosed with behavioural disorders, typically at a younger age compared to mental disorders, such as depression and anxiety, which are more prevalent in girls [14–16]. Kong et al. investigated possible sex differences in cases of pre-existing diabetes or GDM exposure and ADHD and ASD among offspring. They found these disorders to be more prevalent in boys, similar to our findings, but they found no sex differences based on the exposure status within these specific categories [42]. This was also the case in our study concerning all of the examined subcategories. In our study, however, the similarity between sexes in the subcategories might be related to the small number of cases included in them.

Prior meta-analyses have revealed that both maternal pre-existing diabetes and GDM are associated with the risk of ADHD and ASD in offspring [31, 32, 53]. We found a higher prevalence of these disorders in GDM-exposed children compared to controls, but the difference between study groups was statistically insignificant after adjustments. In the univariate analyses, the most significant confounding factors (*p* < 0.001) were maternal BMI (< 18.5 or ≥ 30 kg/m^2^), maternal age (< 25 or 30 to 34 years), adverse perinatal outcomes of the child, maternal smoking and maternal mental health diagnoses. The ability to adjust for these confounders was one of the strengths of our study. A large multinational cohort study investigating GDM exposure as a risk factor for ADHD reached a similar conclusion in a sibship design, suggesting that shared familial factors have a greater impact on ADHD risk than GDM exposure [54]. On the other hand, a subsequent meta-analysis by Rowland and Wilson (2021) did not confirm GDM exposure as a risk factor for ADHD [33].

Additionally, we explored several subcategories of mental and behavioural disorders that have received less attention in the literature. In a study by Kong et al. (2018), GDM exposure showed no increased risk in any of the specific subcategories, which may be related to differences in how the categories were defined and stratified by BMI in their analysis [21]. A large Danish register-based study extensively explored psychiatric disorders (ICD-10: F00–F99) and their subcategories in offspring exposed to GDM up until adulthood [23]. In their analyses, which adjusted for multiple confounders, GDM exposure was identified as an independent risk factor for any psychiatric disorder, anxiety disorders and developmental disorders. Notably, the study also adjusted for paternal psychiatric disorders. Regarding the subcategories, GDM exposure revealed different patterns in our study, where it was an independent risk factor for offspring behavioural disorders, developmental disorders and behavioural disorders with physiological disturbances. This discrepancy may be attributable to the younger age of our cohort and the lower prevalence of GDM associated with the different screening policy in their cohort. Interestingly, their sibship design found no effect of GDM on the risk of any psychiatric disorder, although pre-pregnancy diabetes did exhibit an effect in this design [23].

We conducted a separate analysis of children exposed to insulin-treated and diet-treated GDM, revealing risks for these disorders in both groups compared to controls. Due to the limitations in sample size, these analyses were only conducted for these disorders as a whole. Previous research has suggested that the severity of maternal GDM may correlate especially with the risk of ASD and ADHD in offspring. Specifically, early-onset GDM has been linked to an increased risk of ASD, while GDM requiring medication has been associated with an increased risk of ADHD, in offspring [33]. We did not have data on the time of GDM diagnosis, maternal blood glucose levels or possible hypoglycaemia during pregnancy. A link between insulin therapy-related hypoglycaemia during pregnancy and neurocognitive development of the offspring has also been suspected [22].

Our study had several strengths, the most important of which was that the population-based data we used were comprehensively screened for GDM. The guidelines for screening, diagnosing and treating GDM in the study cohort were also uniform [55]. The proportion of GDM-exposed children in our study was 11.2%, which is comparable to the worldwide prevalence of GDM [3]. The Finnish national registers we employed and the accuracy of GDM diagnosis were both validated [37, 56]. In addition, we had information on many important confounding factors, such as maternal mental health disorders and SES, as, with some of the investigated disorders, heritability plays an important role, particularly ADHD and ASD (average 0.8 for both), and lower SES is known to increase the risk of adverse mental health. In addition, the response to GDM exposure might be sex-dependent, as boys appear to be more sensitive to the effects of adverse prenatal environment than girls [57–61]. The overall prevalence of mental and behavioural disorders in our data (12%) was comparable to the estimate of their worldwide prevalence in children and adolescents [9, 12, 52]. In Finland, access to health care is good, thanks to comprehensive, free and high-quality maternity and child health clinics [62, 63].

The limitations of our study were mainly related to the use of register-based data. As deaths occurring after the perinatal period and emigration were not captured in the registers we employed, some children were lost to follow-up before 10 years of age. However, we have no reason to expect significant differences in deaths or emigration between GDM-exposed children and controls. Additionally, the diagnoses of the examined disorders were retrieved from the FCR, which only contains data on diagnoses in specialised health care. Although mental and behavioural disorders in children are mostly diagnosed in specialised health care in Finland, diagnosis in primary health care is also possible [16, 64, 65]. Another limitation is that although childhood obesity is associated with a higher prevalence of mental disorders [66], our register-based data could not capture this association. Further, the environment in which children grow up is complex and influenced by many factors, including parental drug or alcohol abuse, paternal SES, or family income level. Unfortunately, we could not assess these factors based on our data, nor did we have the data needed to assess the developmental environment after pregnancy. Lastly, although our study demonstrated the association between GDM exposure and childhood mental and behavioural disorders, we could not conclusively demonstrate causation between these factors.

## Conclusion

We found that children exposed to maternal GDM are more likely to have mental and behavioural disorders than unexposed children. However, when the data were stratified by sex, GDM was shown to be an independent risk factor for these disorders only in boys. Sex differences in the effect of GDM exposure on childhood neurodevelopment is an important area for future research. Overall, the risk factors for mental and behavioural disorders are diverse, as prenatal, genetic and environmental factors all play roles. Our study supports the existing evidence that GDM exposure affects the neurodevelopment of children. Furthermore, it extends this evidence by demonstrating that the association is present across a wide range of mental and behavioural disorders in children. In clinical practice, it is important to emphasise preventive actions concerning GDM to promote the health of women and their children.

## Data Availability

The data of the present study are not publicly available because access to the registry data requires permission from the registry authorities. The registers used in this study are maintained by the THL. Researchers can use similar register data from Findata, the Finnish Social and Health Data Permit Authority (https://findata.fi/en/).
